# Event-Triggered ESO-Based Prescribed-Time Funnel Control for Robust Trajectory Tracking of Micro Quadrotor UAVs

**DOI:** 10.3390/mi17060716

**Published:** 2026-06-12

**Authors:** Bofei Wang, Shengsheng Wei, Junqiang Wang

**Affiliations:** 1 Shanxi Key Laboratory of Graphene Sensing Materials and Devices, North University of China, Taiyuan 030051, China; sz202306160@st.nuc.edu.cn; 2 Academy for Advanced Interdisciplinary Research, North University of China, Taiyuan 030051, China; 3 School of Instrument and Electronics, North University of China, Taiyuan 030051, China; 4 School of Semiconductors and Physics, North University of China, Taiyuan 030051, China

**Keywords:** micro quadrotor UAV, PTFC, event-triggered control, ESO, disturbance rejection, trajectory tracking

## Abstract

Micro quadrotor unmanned aerial vehicles (UAVs) are highly sensitive to external disturbances and model uncertainties because of their small mass, low moment of inertia, and limited onboard computational resources. To improve the disturbance rejection and trajectory tracking performance of micro quadrotor UAVs, this paper proposes an event-triggered extended state observer (ET-ESO)-based prescribed-time funnel control (PTFC) method. First, a control-oriented dynamic model of the micro quadrotor is established, in which wind disturbances, unmodeled aerodynamic effects, damping uncertainties, and parameter perturbations are represented as lumped disturbances in the translational and rotational subsystems. Then, two event-triggered ESOs are designed to estimate the lumped disturbances of the velocity and angular velocity channels. Compared with conventional continuously sampled ESO schemes, the proposed event-triggered mechanism reduces the frequency of sensor-to-controller information transmission while preserving disturbance estimation capability. Furthermore, a prescribed-time funnel control law is developed to constrain the position and attitude tracking errors within predefined performance boundaries and ensure convergence to the desired accuracy region within a user-specified time. Lyapunov-based stability analysis is provided to prove the boundedness of all closed-loop signals and the validity of the prescribed funnel constraints. Finally, MATLAB/Simulink simulations based on the Parrot Mambo mini-drone parameters are conducted to verify the effectiveness of the proposed method. The results demonstrate that the proposed controller achieves robust trajectory tracking, effective disturbance compensation, improved transient performance, and reduced control update frequency.

## 1. Introduction

Micro quadrotor unmanned aerial vehicles (UAVs) [1,2,3] have attracted increasing attention in recent years due to their compact structure, high maneuverability, vertical take-off and landing capability, flexible deployment, and suitability for close-range low-altitude flight. Owing to these advantages, they have been widely investigated and applied in indoor inspection, narrow-space exploration, environmental monitoring, disaster search and rescue, target tracking, and cooperative aerial missions. Compared with conventional quadrotor UAVs, micro quadrotors usually have smaller mass, lower moment of inertia, and more limited onboard computational resources. As a result, their flight dynamics are more sensitive to external wind disturbances, unmodeled aerodynamic effects, parametric perturbations, and sensor noise [4]. These uncertainties may not only degrade the trajectory tracking accuracy but also lead to large transient overshoots and long convergence processes. In practical missions such as indoor obstacle avoidance, window crossing, and narrow-space inspection, merely guaranteeing asymptotic stability is insufficient. Therefore, achieving disturbance rejection, transient performance constraint, and prescribed-time error convergence under limited onboard resources remains a challenging issue for micro quadrotor UAVs.

To date, numerous advanced disturbance rejection control strategies have been developed to improve the stability and trajectory tracking performance of micro quadrotor UAVs under external disturbances and model uncertainties [4]. Among them, neural network-based control [5,6,7] and fuzzy approximator-based control [8,9] have been widely investigated due to their strong capability in approximating unknown nonlinear dynamics. For instance, Liu et al. proposed an anti-saturation adaptive finite-time neural network-based fault-tolerant tracking control method for quadrotor UAVs subject to external disturbances, parametric uncertainties, actuator faults, and input saturation [5]. Yang et al. developed a fuzzy approximation-based adaptive finite-time tracking control scheme for quadrotor UAVs with actuator faults, where fuzzy logic systems were used to deal with unknown nonlinearities [9]. Although these function approximator-based methods can effectively estimate or compensate for system uncertainties, they usually involve a large number of adaptive parameters, complex controller structures, and relatively high computational costs. These limitations may become more evident for micro quadrotor UAVs, whose onboard processors, energy supply, and communication resources are generally limited.

To reduce the computational burden caused by neural networks and fuzzy approximators, Han proposed the extended state observer (ESO) method [10]. Compared with function approximation-based methods, the ESO [11] has a simpler structure and mainly relies on a bandwidth tuning parameter, making it suitable for estimating lumped disturbances in uncertain nonlinear systems. Owing to these advantages, ESO-based and active disturbance rejection control (ADRC)-based methods have been applied to quadrotor UAV trajectory tracking and disturbance compensation [12,13,14]. For example, Xu et al. proposed an event-triggered cascade ADRC scheme for quadrotor UAV trajectory tracking, which can suppress unknown disturbances and reduce the transmission frequency of sampled data [14]. However, many existing ESO-based control strategies still rely on periodic or continuous state sampling, which increases the sensor-to-controller transmission burden and may cause unnecessary resource consumption. Therefore, it is meaningful to design an ESO scheme based on non-periodic measurement updates, so that system uncertainties can be effectively estimated while reducing the sampling and communication load of micro quadrotor UAVs.

It should also be noted that many existing control methods for micro quadrotor UAVs mainly focus on asymptotic stability or finite-time convergence [4], while the transient and steady-state tracking performance, such as convergence time, overshoot, and tracking error boundaries, cannot be explicitly adjusted in advance. In some finite-time control approaches, the settling time is closely related to the initial tracking error and controller gains. As a result, different initial conditions may lead to different convergence times, and large initial deviations may significantly prolong the stabilization process. This characteristic is not desirable for micro quadrotor UAVs performing time-critical tasks, such as narrow-space flight, fast maneuvering, and precise trajectory tracking.

To address these issues, funnel control (FC) [15] and prescribed performance control (PPC) [13] methods have been proposed to constrain the tracking error within predefined performance boundaries through error transformation. Such methods make it possible to regulate the transient response and steady-state accuracy of the closed-loop system. For example, Lapandić et al. introduced a prescribed performance control framework for robust trajectory tracking of underactuated quadrotors, ensuring that tracking errors remain within designer-specified time-varying performance functions [16]. Tian et al. proposed a prescribed-time fault-tolerant tracking control method for quadrotor UAVs with guaranteed performance, where the position and attitude tracking errors can converge to prescribed accuracy regions within a preselected time [17]. Nevertheless, conventional FC or PPC methods often adopt exponential performance functions, which implies that the tracking errors approach the desired steady-state region only as time tends to infinity. In practical micro quadrotor UAV applications, the convergence time is usually required to be specified according to mission demands. Therefore, developing a prescribed-time FC strategy for micro quadrotor UAVs is of significant importance for simultaneously guaranteeing disturbance rejection, transient performance constraints, and user-defined convergence time.

In addition to observer-based and prescribed-performance-based control strategies, robust model predictive control methods, especially Tube-MPC-based approaches, have recently attracted increasing attention in robust trajectory tracking and maneuver planning problems (see, e.g., [18,19]). Tube-MPC methods usually construct a robust invariant tube around a nominal trajectory, such that the actual system trajectory remains inside the tube under bounded disturbances and model uncertainties. This framework is advantageous for explicitly handling state/input constraints and predictive optimization. Recently, Schitz et al. proposed a robust maneuver planning method with scalable prediction horizons based on a move-blocking strategy, which reduces the computational burden of long-horizon MPC while maintaining robust planning capability [20]. Such methods are important for aerial systems that require safe planning under uncertainty and constraints. However, the online receding-horizon optimization or long-horizon planning process may still increase the computational burden, especially for micro quadrotor UAVs with limited onboard resources. Different from Tube-MPC and move-blocking MPC methods, the method proposed in this paper adopts an event-triggered ESO to estimate lumped disturbances and a prescribed-time funnel controller to impose transient and steady-state tracking constraints, without solving an online optimization problem. Therefore, the proposed method provides an observer-based robust tracking alternative with relatively low computational complexity, while MPC-based methods remain attractive when explicit constraint handling, predictive optimization, and robust maneuver planning are required.

Communication latency is another important issue in networked and resource-constrained UAV control systems. Recent studies have shown that network-induced delays, packet dropouts, and communication uncertainties may degrade closed-loop stability and tracking performance (see, e.g., [21,22,23]). Although the event-triggered mechanism adopted in this paper can reduce unnecessary sensor-to-controller transmissions, it does not explicitly compensate for communication latency. Therefore, the present work focuses on reducing the update frequency under non-periodic sampling, while delay compensation and latency-aware event-triggered control will be considered in future work.

Motivated by the above observations, this paper proposes an event-triggered ESO-based prescribed-time funnel control for robust trajectory tracking of micro quadrotor UAVs. The basic idea is to introduce an event-triggered extended state observer (ET-ESO) into the micro quadrotor control framework to estimate and compensate for lumped disturbances in both translational and rotational subsystems. Meanwhile, a prescribed-time funnel control (PTFC) is constructed to constrain the trajectory tracking errors within predefined boundaries and guarantee their convergence to desired accuracy regions within a user-specified time. Compared with disturbance compensation schemes relying on neural networks or function approximators, the proposed event-triggered ESO has a simpler observer structure and mainly requires bandwidth-parameter tuning, indicating its potential applicability to resource-constrained micro quadrotor platforms. Compared with conventional continuously sampled ESO-based control methods, the fixed-threshold event-triggered mechanism reduces the update frequency of state information from the sensing side to the control side while maintaining disturbance estimation and trajectory tracking performance. Furthermore, compared with asymptotic convergence control and traditional prescribed performance control methods, the proposed prescribed-time funnel control scheme explicitly specifies the tracking error boundary, steady-state accuracy, and convergence time, thereby improving both transient and steady-state tracking performance.

It should be emphasized that the proposed method is not a direct combination of event-triggered ESO and prescribed-time control. When the ESO is implemented with non-periodic event-triggered measurements, sampling-induced errors are introduced into the observer error dynamics. Meanwhile, the prescribed-time funnel controller requires the tracking errors to remain strictly inside predefined time-varying boundaries. Therefore, the residual disturbance-estimation errors caused by event-triggered updates must be explicitly incorporated into the funnel stability analysis. The theoretical challenge addressed in this paper is to establish a coupled closed-loop stability result that simultaneously guarantees practical disturbance estimation under non-periodic measurement updates, boundedness of the transformed funnel errors, and prescribed-time tracking performance for both translational and rotational subsystems.

The main contributions of this paper are summarized as follows:

1. A control-oriented dynamic model of the micro quadrotor UAV is established, where wind disturbances, unmodeled aerodynamic effects, damping uncertainties, and mass/inertia perturbations are represented as lumped disturbances in the translational and rotational subsystems.
2. An ET-ESO is designed to estimate the lumped disturbances online. The proposed observer reduces continuous information transmission from the sensing side to the control side while preserving disturbance rejection capability. Different from continuously updated ESO schemes, the event-triggered sampling error is explicitly introduced into the observer error dynamics, and a scaled-error analysis is developed to prove practical disturbance-estimation boundedness under non-periodic measurements.
3. A PTFC law is developed to ensure that the position and attitude tracking errors remain within predefined performance boundaries and converge to the desired steady-state accuracy regions within a prescribed time. In the controller design, the residual ET-ESO estimation errors are considered in the funnel error dynamics, so that the prescribed-time performance constraints can be maintained despite event-triggered disturbance compensation.
4. Lyapunov-based analysis is provided to prove the boundedness of all closed-loop signals, the validity of the funnel constraints, and the stability of the proposed control scheme. The analysis links the non-periodic observer-update mechanism with the prescribed-time funnel transformation, which provides a theoretical guarantee for simultaneous disturbance rejection, reduced information transmission, and prescribed-time convergence. Comparative simulations are conducted to verify the effectiveness of the proposed method in disturbance rejection, transient performance improvement, and control update reduction.

## 2. Preliminaries and Problem Formulation

### 2.1. Micro Quadrotor UAV Modle

**Figure 1:**
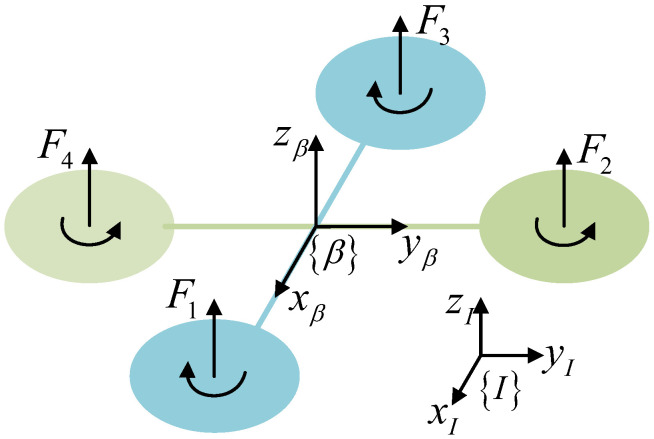
Schematic diagram of micro quadrotors.

To facilitate the analysis and controller design for the micro quadrotor UAV, a body-fixed frame $\left\{\beta \right\}=\left\{x_{\beta },y_{\beta },z_{\beta }\right\}$ and an earth-fixed inertial frame $\left\{I\right\}=\left\{x_{I},y_{I},z_{I}\right\}$ are introduced. As shown in Figure 1, the thrust $F_{m}\;\left(m=1,2,3,4\right)$ generated by the rotation of the corresponding propeller can steer both the position and attitude of the micro quadrotor. Compared with conventional quadrotors, the considered micro quadrotor is more sensitive to external disturbances and model uncertainties due to its small mass, low rotational inertia, and limited disturbance attenuation capability. Resorting to the standard quadrotor formulation, the kinematics and dynamics of the micro quadrotor UAV can be described as

(1) $$\left\{\begin{array}{c} \dot{P}=V,\\ \dot{V}=f_{v}\left(V\right)+F_{v}\left(ℵ,u_{F}\right)+d_{v},\\ \dot{ℵ}=\omega ,\\ \dot{\omega }=f_{\omega }\left(\omega \right)+F_{\omega }\left(u\right)+d_{\omega }, \end{array}\right.$$

where $P={\left[P_{x},P_{y},P_{z}\right]}^{T}$ and $V={\left[V_{x},V_{y},V_{z}\right]}^{T}$ denote the position and linear velocity of the micro quadrotor in the inertial frame {I}, respectively. The vectors $ℵ={\left[{ℵ}_{\phi },{ℵ}_{\theta },{ℵ}_{\psi }\right]}^{T}$ and $\omega ={\left[\omega_{\phi },\omega_{\theta },\omega_{\psi }\right]}^{T}$ represent the attitude angle and angular velocity of the micro quadrotor, respectively, where ${ℵ}_{\phi }$, ${ℵ}_{\theta }$, and ${ℵ}_{\psi }$ are the roll, pitch, and yaw angles.

**Figure 2:**
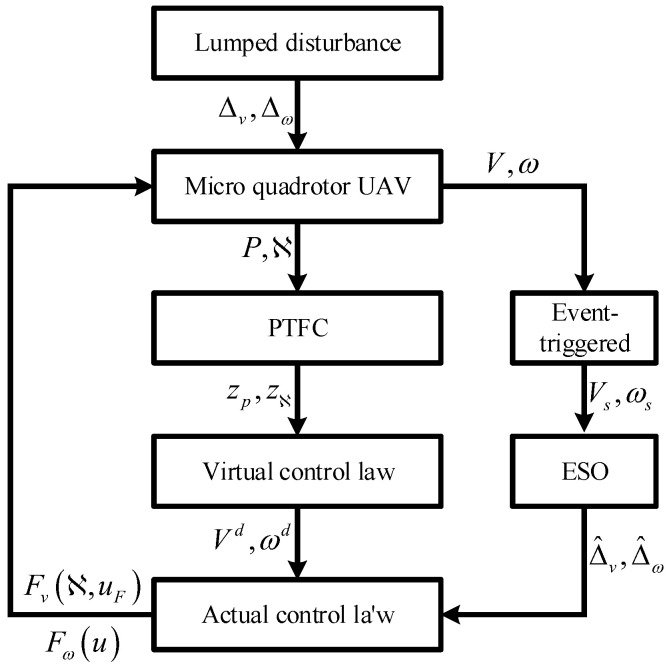
Architecture of the proposed algorithm.

In the above model, $F_{v}\left(ℵ,u_{F}\right)$ and $F_{\omega }\left(u\right)$ denote the translational and rotational control input terms, respectively, while $d_{v}$ and $d_{\omega }$ represent the unknown disturbance terms in the translational and rotational subsystems. For the considered micro quadrotor UAV, the disturbance terms are described as

(2) $$\left\{\begin{array}{c} d_{v}=d_{v,w}+d_{v,a}+d_{v,\Delta }+d_{v,m},\\ d_{\omega }=d_{\omega ,w}+d_{\omega ,a}+d_{\omega ,\Delta }+d_{\omega ,J}, \end{array}\right.$$

where $d_{v,w}$ and $d_{\omega ,w}$ denote the wind-induced force and torque disturbances, $d_{v,a}$ and $d_{\omega ,a}$ denote the unmodeled aerodynamic force and moment, $d_{v,\Delta }$ and $d_{\omega ,\Delta }$ represent the uncertainties caused by translational and rotational damping variations, and $d_{v,m}$ and $d_{\omega ,J}$ are associated with mass and inertia perturbations, respectively. Compared with conventional quadrotors, these disturbances have more pronounced effects on micro quadrotor UAVs because of their small mass and low moment of inertia. Based on the above micro quadrotor model, the overall architecture of the proposed control strategy is illustrated in Figure 2.

### 2.2. Event-Triggered Extended State Observer

In this subsection, two ET-ESOs are designed to estimate the lumped disturbances in the translational and rotational subsystems while reducing the sensor-to-controller transmission burden. For controller design, the micro quadrotor dynamics are rewritten as

(3) $$\left\{\begin{array}{c} \dot{P}=V,\\ \dot{V}=F_{v}\left(ℵ,u_{F}\right)+\Delta_{v},\\ \dot{ℵ}=\omega ,\\ \dot{\omega }=F_{\omega }\left(u\right)+\Delta_{\omega }, \end{array}\right.$$

where

(4) $$\Delta_{v}=f_{v}\left(V\right)+d_{v},$$

(5) $$\Delta_{\omega }=f_{\omega }\left(\omega \right)+d_{\omega }$$

represent the total lumped disturbances in the translational and rotational subsystems, respectively.

Following the classical ESO [24] design principle, $\Delta_{v}$ and $\Delta_{\omega }$ are treated as augmented states. To reduce continuous signal transmission from sensors to the controller, the latest triggered velocity and angular velocity signals are used in the observer. The proposed ET-ESOs are constructed as

(6) $$\left\{\begin{array}{c} \dot{\hat{V}}=F_{v}\left(ℵ,u_{F}\right)+{\hat{\Delta }}_{v}+2\varpi \left(V_{s}-\hat{V}\right),\\ {\dot{\hat{\Delta }}}_{v}=\varpi^{2}\left(V_{s}-\hat{V}\right), \end{array}\right.$$

and

(7) $$\left\{\begin{array}{c} \dot{\hat{\omega }}=F_{\omega }\left(u\right)+{\hat{\Delta }}_{\omega }+2\gamma \left(\omega_{s}-\hat{\omega }\right),\\ {\dot{\hat{\Delta }}}_{\omega }=\gamma^{2}\left(\omega_{s}-\hat{\omega }\right), \end{array}\right.$$

where $\hat{V}$ and $\hat{\omega }$ are the estimates of *V* and ω, respectively. The terms ${\hat{\Delta }}_{v}$ and ${\hat{\Delta }}_{\omega }$ denote the estimates of the translational and rotational lumped disturbances. The positive constants ϖ and γ are the observer bandwidth parameters. The signals $V_{s}$, $\omega_{s}$, and ${ℵ}_{s}$ denote the latest event-triggered sampled values of *V*, ω, and *ℵ*, respectively.

The event-triggered sampling rule for the velocity signal is defined as

(8) $$\left\{\begin{array}{c} V_{s}\left(t\right)=V\left(t_{k}^{v}\right),\;t\in \left[t_{k}^{v},t_{k+1}^{v}\right),\\ e_{V}\left(t\right)=V_{s}\left(t\right)-V\left(t\right),\\ t_{k+1}^{v}=\inf\left\{t>t_{k}^{v}:∥e_{V}\left(t\right)∥\geq \delta_{v}\right\}, \end{array}\right.$$

where $\delta_{v}>0$ is the velocity triggering threshold.

Similarly, the event-triggered sampling rule for the angular velocity signal is given by

(9) $$\left\{\begin{array}{c} \omega_{s}\left(t\right)=\omega \left(t_{k}^{\omega }\right),\;t\in \left[t_{k}^{\omega },t_{k+1}^{\omega }\right),\\ e_{\omega }\left(t\right)=\omega_{s}\left(t\right)-\omega \left(t\right),\\ t_{k+1}^{\omega }=\inf\left\{t>t_{k}^{\omega }:∥e_{\omega }\left(t\right)∥\geq \delta_{\omega }\right\}, \end{array}\right.$$

where $\delta_{\omega }>0$ is the angular velocity triggering threshold.

Next, the estimation feasibility of the proposed ET-ESOs is analyzed. For compactness, the translational and rotational subsystems are written in a unified form as

(10) $${\dot{q}}_{i}=U_{i}+\Delta_{i},\;i\in \left\{v,\omega \right\},$$

where

(11) $$q_{v}=V,\;U_{v}=F_{v}\left(ℵ,u_{F}\right),\;\Delta_{v}=f_{v}\left(V\right)+d_{v},$$

and

(12) $$q_{\omega }=\omega ,\;U_{\omega }=F_{\omega }\left(u\right),\;\Delta_{\omega }=f_{\omega }\left(\omega \right)+d_{\omega }.$$

Here, $\Delta_{v}$ and $\Delta_{\omega }$ denote the total lumped disturbances in the translational and rotational subsystems, respectively.

The event-triggered sampled signal is defined as

(13) $$\left\{\begin{array}{c} q_{i,s}\left(t\right)=q_{i}\left(t_{k}^{i}\right),\;t\in \left[t_{k}^{i},t_{k+1}^{i}\right),\\ e_{i,s}\left(t\right)=q_{i,s}\left(t\right)-q_{i}\left(t\right),\\ t_{k+1}^{i}=\inf\left\{t>t_{k}^{i}:∥e_{i,s}\left(t\right)∥\geq h_{i}^{-2}\sigma_{i}\right\}, \end{array}\right.$$

where $q_{i,s}\left(t\right)$ is the latest transmitted signal, $e_{i,s}\left(t\right)$ is the sampling error, $h_{i}$ is the observer bandwidth, and $\sigma_{i}>0$ is a fixed triggering parameter. Specifically, for the translational subsystem, $h_{v}=\varpi$ and $\sigma_{v}=\zeta$; for the rotational subsystem, $h_{\omega }=\gamma$ and $\sigma_{\omega }=\xi$.

Based on the sampled signal $q_{i,s}\left(t\right)$, the ET-ESO is designed as

(14) $$\left\{\begin{array}{c} {\dot{\hat{q}}}_{i}=U_{i}+{\hat{\Delta }}_{i}+2h_{i}\left(q_{i,s}-{\hat{q}}_{i}\right),\\ {\dot{\hat{\Delta }}}_{i}=h_{i}^{2}\left(q_{i,s}-{\hat{q}}_{i}\right), \end{array}\right.$$

where ${\hat{q}}_{i}$ and ${\hat{\Delta }}_{i}$ are the estimates of $q_{i}$ and $\Delta_{i}$, respectively.

**Assumption 1.**  *The lumped disturbances $\Delta_{j}$ and their time derivatives are bounded, namely*

(15) $$∥\Delta_{j}∥\;\leq \;{\bar{\Delta }}_{j},\;∥{\dot{\Delta }}_{j}∥\leq L_{j},\;j\in \left\{v,\omega \right\},$$

*where ${\bar{\Delta }}_{j}$ and $L_{j}$ are unknown positive constants.*

**Remark 1.**  *Assumption 1 is a standard condition in ESO-based control design. For micro quadrotor UAVs, the lumped disturbances mainly originate from external wind disturbances, unmodeled aerodynamic effects, damping uncertainties, and parameter perturbations. Since these disturbances are physically bounded in practical flight conditions, Assumption 1 does not impose a restrictive requirement on the proposed control scheme.*

Define the estimation errors as

(16) $${\tilde{\eta }}_{j}=\eta_{j}-{\hat{\eta }}_{j},\;{\tilde{\Delta }}_{j}=\Delta_{j}-{\hat{\Delta }}_{j},\;j\in \left\{v,\omega \right\}.$$

Recalling (10) and (14), the estimation error dynamics can be obtained as

(17) $$\left\{\begin{array}{c} {\dot{\tilde{\eta }}}_{j}=-2h_{j}{\tilde{\eta }}_{j}+{\tilde{\Delta }}_{j}-2h_{j}e_{j,s},\\ {\dot{\tilde{\Delta }}}_{j}=-h_{j}^{2}{\tilde{\eta }}_{j}-h_{j}^{2}e_{j,s}+{\dot{\Delta }}_{j}. \end{array}\right.$$

where $\eta_{v}=V$, $\eta_{\omega }=\omega$, $h_{v}=\varpi$, and $h_{\omega }=\gamma$.

To analyze the estimation performance, introduce the scaled estimation errors

(18) $$\chi_{j1}=h_{j}{\tilde{\eta }}_{j},\;\chi_{j2}={\tilde{\Delta }}_{j},$$

and the scaled sampling error

(19) $$\chi_{j*}=h_{j}e_{j,s}.$$

Let $\tau =h_{j}t$. Then, from (17), one has

(20) $$\left\{\begin{array}{c} \frac{d\chi_{j1}}{d\tau }=\chi_{j2}-2\chi_{j1}-2\chi_{j*},\\ \frac{d\chi_{j2}}{d\tau }=-\chi_{j1}-\chi_{j*}+h_{j}^{-1}{\dot{\Delta }}_{j}. \end{array}\right.$$

According to the event-triggered sampling rule (13), it follows that

(21) $$∥e_{j,s}∥\leq h_{j}^{-2}\sigma_{j},$$

and therefore

(22) $$∥\chi_{j*}∥\leq h_{j}^{-1}\sigma_{j}.$$

Here, $\sigma_{v}=\zeta$ and $\sigma_{\omega }=\xi$.

The scaled error dynamics (20) can be rewritten as

(23) $$\frac{d\chi_{j}}{d\tau }=A\chi_{j}+\varrho_{j},$$

where

(24) $$\chi_{j}=\left[\begin{array}{c} \chi_{j1}\\ \chi_{j2} \end{array}\right],\;A=A⊗I_{3},\;A=\left[\begin{array}{cc} -2 & 1\\ -1 & 0 \end{array}\right],$$

and

(25) $$\varrho_{j}=\left[\begin{array}{c} -2\chi_{j*}\\ -\chi_{j*}+h_{j}^{-1}{\dot{\Delta }}_{j} \end{array}\right].$$

Since *A* is Hurwitz, A is also Hurwitz. Thus, for any positive definite matrix $Q_{j}$, there exists a positive definite matrix $P_{j}$ satisfying

(26) $$A^{T}P_{j}+P_{j}A=-Q_{j}.$$

Choose the Lyapunov function

(27) $$V_{j}=\chi_{j}^{T}P_{j}\chi_{j}.$$

Then,

(28) $$\lambda_{j1}∥\chi_{j}{∥}^{2}\leq V_{j}\leq \lambda_{j2}{∥\chi_{j}∥}^{2},$$

where $\lambda_{j1}=\lambda_{\min}\left(P_{j}\right)$ and $\lambda_{j2}=\lambda_{\max}\left(P_{j}\right)$.

Differentiating $V_{j}$ along (23) yields

(29) $$\begin{array}{cc} \frac{dV_{j}}{d\tau } & =\chi_{j}^{T}\left(A^{T}P_{j}+P_{j}A\right)\chi_{j}+2\chi_{j}^{T}P_{j}\varrho_{j}\\ & =-\chi_{j}^{T}Q_{j}\chi_{j}+2\chi_{j}^{T}P_{j}\varrho_{j}. \end{array}$$

Using (25), (15) and (22), one obtains

(30) $$∥\varrho_{j}∥\leq \frac{c_{j}}{h_{j}},$$

where

(31) $$c_{j}=3\sigma_{j}+L_{j}.$$

Here, $L_{j}$ denotes the upper bound of $∥{\dot{\Delta }}_{j}∥$ in Assumption 1.

Thus,

(32) $$\begin{array}{cc} \frac{dV_{j}}{d\tau } & \leq -\lambda_{j3}{∥\chi_{j}∥}^{2}+2∥P_{j}∥∥\chi_{j}∥∥\varrho_{j}∥\\ & \leq -\frac{\lambda_{j3}}{\lambda_{j2}}V_{j}+\frac{2∥P_{j}∥c_{j}}{h_{j}\sqrt{\lambda_{j1}}}\sqrt{V_{j}}, \end{array}$$

where $\lambda_{j3}=\lambda_{\min}\left(Q_{j}\right)$.

Let $y_{j}=\sqrt{V_{j}}$. From (32), it follows that

(33) $$\frac{dy_{j}}{d\tau }\leq -\frac{\lambda_{j3}}{2\lambda_{j2}}y_{j}+\frac{∥P_{j}∥c_{j}}{h_{j}\sqrt{\lambda_{j1}}}.$$

Solving the above inequality gives

(34) $$y_{j}\left(\tau \right)\leq y_{j}\left(0\right)e^{-\frac{\lambda_{j3}}{2\lambda_{j2}}\tau }+\frac{2\lambda_{j2}∥P_{j}∥c_{j}}{h_{j}\lambda_{j3}\sqrt{\lambda_{j1}}}.$$

Together with (28), one has

(35) $$∥\chi_{j}\left(\tau \right)∥\leq \sqrt{\frac{\lambda_{j2}}{\lambda_{j1}}}∥\chi_{j}\left(0\right)∥e^{-\frac{\lambda_{j3}}{2\lambda_{j2}}\tau }+\frac{2\lambda_{j2}∥P_{j}∥c_{j}}{h_{j}\lambda_{j1}\lambda_{j3}}.$$

Since ${\tilde{\eta }}_{j}=h_{j}^{-1}\chi_{j1}$ and ${\tilde{\Delta }}_{j}=\chi_{j2}$, the estimation errors satisfy

(36) $$∥{\tilde{\eta }}_{j}∥\leq h_{j}^{-1}∥\chi_{j}∥,\;∥{\tilde{\Delta }}_{j}∥\leq ∥\chi_{j}∥.$$

Furthermore, for

(37) $$t\geq \frac{4\lambda_{j2}\ln h_{j}}{\lambda_{j3}h_{j}},$$

the exponential term in (35) can be reduced to the order of $h_{j}^{-1}$. Therefore, there exists a positive constant $\Theta_{j}$ such that

(38) $$∥{\tilde{\eta }}_{j}∥\leq \Theta_{j}h_{j}^{-2},\;∥{\tilde{\Delta }}_{j}∥\leq \Theta_{j}h_{j}^{-1},$$

where

(39) $$\Theta_{j}=\sqrt{\frac{\lambda_{j2}}{\lambda_{j1}}}∥\left[\begin{array}{c} {\tilde{\eta }}_{j}\left(0\right)\\ {\tilde{\Delta }}_{j}\left(0\right) \end{array}\right]∥+\frac{2\lambda_{j2}∥P_{j}∥c_{j}}{\lambda_{j1}\lambda_{j3}}.$$

**Remark 2.**  *The bound in the above equation is well defined and does not contain any division by the disturbance estimation error ${\tilde{\Delta }}_{j}$. Therefore, no singularity is caused when ${\tilde{\Delta }}_{j}$ is zero or very small; this case only indicates that the ET-ESO has achieved a small estimation error. The denominators of the bound are determined by $\lambda_{j1}=\lambda_{\min}\left(P_{j}\right)$ and $\lambda_{j3}=\lambda_{\min}\left(Q_{j}\right)$, where $P_{j}$ and $Q_{j}$ are positive definite matrices. Hence, the observer error bound remains finite as long as $h_{j}>0$ and the Lyapunov equation is constructed with positive definite matrices. In implementation, the triggering parameter $\sigma_{j}$ should not be selected excessively small, because an overly small threshold may cause frequent triggering and noise sensitivity. In addition, the prescribed-time funnel transformation should be implemented with a safety margin to avoid numerical ill-conditioning when $|e_{i}\left(t\right)|$ approaches $\rho_{i}\left(t\right)$. A practical choice is to require $|e_{i}\left(t\right)|\leq \left(1-\varepsilon \right)\rho_{i}\left(t\right)$ with a small ε>0, or to use a saturated/regularized transformation near the funnel boundary.*

Consequently, the estimation errors of the proposed ET-ESO are bounded and practically convergent. Moreover, by increasing the observer bandwidth $h_{j}$, the estimation errors can be made arbitrarily small in the sense of (38). This completes the proof.

For the translational ET-ESO, one has

(40) $$∥V-\hat{V}∥\leq \Theta_{v}\varpi^{-2},\;∥\Delta_{v}-{\hat{\Delta }}_{v}∥\leq \Theta_{v}\varpi^{-1}.$$

For the rotational ET-ESO, one has

(41) $$∥\omega -\hat{\omega }∥\leq \Theta_{\omega }\gamma^{-2},\;∥\Delta_{\omega }-{\hat{\Delta }}_{\omega }∥\leq \Theta_{\omega }\gamma^{-1}.$$

### 2.3. Prescribed-Time Funnel Control

To guarantee the transient and steady-state tracking performance of the micro quadrotor UAV, a PTFC strategy is introduced in this subsection. Define the controlled output vector as $q={\left[P^{T},{ℵ}^{T}\right]}^{T}={\left[P_{x},P_{y},P_{z},{ℵ}_{\phi },{ℵ}_{\theta },{ℵ}_{\psi }\right]}^{T}$, where $P={\left[P_{x},P_{y},P_{z}\right]}^{T}$ is the position vector and $ℵ={\left[{ℵ}_{\phi },{ℵ}_{\theta },{ℵ}_{\psi }\right]}^{T}$ is the attitude angle vector. Let $q_{d}={\left[P_{d}^{T},{ℵ}_{d}^{T}\right]}^{T}$ be the desired reference trajectory. The tracking error of the *i*-th controlled channel is defined as $e_{i}\left(t\right)=q_{i}\left(t\right)-q_{d,i}\left(t\right)$, i=1,2,…,6.

The objective of the prescribed-time funnel control is to ensure that the tracking error evolves within a predefined performance boundary, i.e., $-\rho_{i}\left(t\right)<e_{i}\left(t\right)<\rho_{i}\left(t\right)$ for all t≥0, where $\rho_{i}\left(t\right)$ is the prescribed-time performance function. In this paper, $\rho_{i}\left(t\right)$ is designed as

(42) $$\rho_{i}\left(t\right)=\left\{\begin{array}{cc} \rho_{i,\infty }+\left(\rho_{i,0}-\rho_{i,\infty }\right)\kappa_{i}\left(t\right),\; & 0\leq t<T_{i},\\ \rho_{i,\infty },\; & t\geq T_{i}, \end{array}\right.$$

where $\rho_{i,0}>\rho_{i,\infty }>0$, $T_{i}>0$ is the user-specified convergence time, and

$$\kappa_{i}\left(t\right)=1-10{\left(\frac{t}{T_{i}}\right)}^{3}+15{\left(\frac{t}{T_{i}}\right)}^{4}-6{\left(\frac{t}{T_{i}}\right)}^{5},\;0\leq t<T_{i}.$$

It can be verified that $\kappa_{i}\left(0\right)=1$, $\kappa_{i}\left(T_{i}\right)=0$, and ${\dot{\kappa }}_{i}\left(t\right)=-30T_{i}^{-1}{\left(t/T_{i}\right)}^{2}{\left(1-t/T_{i}\right)}^{2}\leq 0$. Therefore, $\rho_{i}\left(t\right)$ is positive and monotonically decreasing on $\left[0,T_{i}\right)$ and remains equal to $\rho_{i,\infty }$ for $t\geq T_{i}$. The parameter $\rho_{i,0}$ determines the allowable initial tracking error and transient overshoot, while $\rho_{i,\infty }$ specifies the maximum steady-state tracking error after the prescribed time $T_{i}$.

To avoid the non-differentiability problem caused by conventional funnel error transformations involving $|e_{i}|$, a smooth funnel error transformation is introduced as

(43) $$z_{i}=\frac{e_{i}}{\sqrt{\rho_{i}^{2}\left(t\right)-e_{i}^{2}}},\;i=1,2,\ldots ,6.$$

This transformation is well defined if the initial condition satisfies $\left|e_{i}\left(0\right)\right|<\rho_{i}\left(0\right)$. Moreover, when $\left|e_{i}\left(t\right)\right|$ approaches $\rho_{i}\left(t\right)$, the transformed error $z_{i}$ tends to infinity. Therefore, keeping $z_{i}$ bounded guarantees that the original tracking error $e_{i}\left(t\right)$ remains strictly inside the prescribed funnel boundary.

Differentiating (43) yields

(44) $${\dot{z}}_{i}=M_{i}\left({\dot{e}}_{i}-\frac{{\dot{\rho }}_{i}}{\rho_{i}}e_{i}\right),\;M_{i}=\frac{\rho_{i}^{2}}{{\left(\rho_{i}^{2}-e_{i}^{2}\right)}^{3/2}}>0.$$

For the position subsystem, the tracking error is $e_{P}=P-P_{d}$, with ${\dot{e}}_{P}=V-{\dot{P}}_{d}$. For the attitude subsystem, the tracking error is $e_{ℵ}=ℵ-{ℵ}_{d}$. Since $\dot{ℵ}=\omega$, one has ${\dot{e}}_{ℵ}=\omega -{\dot{ℵ}}_{d}$.

### 2.4. Controller Design

For compact controller design, the micro quadrotor UAV dynamics are rewritten in the following unified second-order form:

(45) $$\left\{\begin{array}{c} {\dot{q}}_{i}=\nu_{i},\\ {\dot{\nu }}_{i}=U_{i}+\Delta_{i}, \end{array}\right.\;i=1,2,\ldots ,6,$$

where $q={\left[P^{T},{ℵ}^{T}\right]}^{T}$ and $\nu ={\left[V^{T},\omega^{T}\right]}^{T}$. For i=1,2,3, $q_{i}$ represents the position components $P_{x}$, $P_{y}$, and $P_{z}$, while for i=4,5,6, $q_{i}$ represents the attitude angle components ${ℵ}_{\phi }$, ${ℵ}_{\theta }$, and ${ℵ}_{\psi }$. The term $U_{i}$ denotes the corresponding control input, and $\Delta_{i}$ is the lumped disturbance estimated by the ET-ESO.

*Step 1: Prescribed-time funnel transformation.*

Using the prescribed-time funnel transformation in (44), the transformed tracking error dynamics can be obtained as

$${\dot{z}}_{i}=M_{i}\left(\nu_{i}-{\dot{q}}_{d,i}-\frac{{\dot{\rho }}_{i}}{\rho_{i}}e_{i}\right).$$

*Step 2: Virtual control law.*

To stabilize the funnel transformed error, the virtual velocity command is designed as

(46) $$\alpha_{i}=-k_{q,i}M_{i}^{-1}z_{i}+{\dot{q}}_{d,i}+\frac{{\dot{\rho }}_{i}}{\rho_{i}}e_{i},$$

where $k_{q,i}>0$ is the funnel control gain. Define the velocity tracking error as $s_{i}={\hat{\nu }}_{i}-\alpha_{i}$, where ${\hat{\nu }}_{i}$ is the velocity or angular velocity estimate generated by the ET-ESO, and define the estimation error as ${\tilde{\nu }}_{i}=\nu_{i}-{\hat{\nu }}_{i}$. Substituting (46) into the transformed error dynamics yields

$$\begin{array}{cc} {\dot{z}}_{i} & =M_{i}\left(\nu_{i}-\alpha_{i}-k_{q,i}M_{i}^{-1}z_{i}\right)\\ & =-k_{q,i}z_{i}+M_{i}\left(s_{i}+{\tilde{\nu }}_{i}\right). \end{array}$$

*Step 3: Actual control law.*

According to the ET-ESO, the estimate ${\hat{\nu }}_{i}$ satisfies

$${\dot{\hat{\nu }}}_{i}=U_{i}+{\hat{\Delta }}_{i}+2h_{i}\left(\nu_{i,s}-{\hat{\nu }}_{i}\right),$$

where ${\hat{\Delta }}_{i}$ is the estimated lumped disturbance, $h_{i}$ is the observer bandwidth, and $\nu_{i,s}$ is the latest event-triggered sampled signal. Then, the derivative of $s_{i}$ can be written as

$${\dot{s}}_{i}=U_{i}+{\hat{\Delta }}_{i}+2h_{i}\left(\nu_{i,s}-{\hat{\nu }}_{i}\right)-{\dot{\alpha }}_{i}.$$

The actual control law is designed as

(47) $$U_{i}=-k_{\nu ,i}s_{i}-M_{i}z_{i}-{\hat{\Delta }}_{i}-2h_{i}\left(\nu_{i,s}-{\hat{\nu }}_{i}\right)+{\dot{\alpha }}_{i},$$

where $k_{\nu ,i}>0$ is the velocity error control gain. Substituting (47) into the derivative of $s_{i}$ gives

$${\dot{s}}_{i}=-k_{\nu ,i}s_{i}-M_{i}z_{i}.$$

**Assumption 2.**  *The desired trajectory $q_{d,i}$, the prescribed-time performance function $\rho_{i}\left(t\right)$, and the virtual control signal $\alpha_{i}$ are sufficiently smooth. Moreover, ${\dot{\alpha }}_{i}$ is bounded, i.e., $\left|{\dot{\alpha }}_{i}\right|\leq {\bar{\alpha }}_{i}$, where ${\bar{\alpha }}_{i}$ is a positive constant.*

The stability analysis below is directly associated with the control architecture shown in Figure 2. The event-triggered block provides the sampled velocity and angular velocity signals, and its influence is reflected by the sampling errors in the ET-ESO error dynamics. The ESO block estimates the lumped disturbances in the translational and rotational subsystems, and its estimation error bounds are used in the subsequent closed-loop analysis. The PTFC block and the virtual control law transform the original position and attitude tracking errors into prescribed-time funnel errors and generate the desired velocity/angular velocity commands. The actual control law then uses the ET-ESO disturbance estimates to stabilize the velocity and angular velocity error dynamics. Therefore, the proof is organized according to the information flow in Figure 2: the boundedness of the ET-ESO estimation errors is first established, then the boundedness of the transformed funnel errors and velocity tracking errors is proved, and finally the validity of the prescribed-time funnel constraints for the original tracking errors is obtained.

**Theorem 1.**  *Consider the closed-loop system corresponding to the architecture in Figure 2, which consists of the micro quadrotor UAV dynamics (45), the event-triggered sampling mechanism, the ET-ESO, the prescribed-time funnel transformation, the virtual control law (46), and the actual control law (47). If Assumptions 1–2 hold and the initial tracking error satisfies $|e_{i}\left(0\right)|<\rho_{i}\left(0\right)$, then all closed-loop signals are bounded. Moreover, the tracking error remains within the prescribed-time funnel boundary, i.e., $|e_{i}\left(t\right)|<\rho_{i}\left(t\right)$ for all t≥0.*

**Proof.**  Choose the Lyapunov function candidate as

$$V_{i}=\frac{1}{2}z_{i}^{2}+\frac{1}{2}s_{i}^{2}.$$

Taking its time derivative gives

$$\begin{array}{cc} {\dot{V}}_{i} & =z_{i}{\dot{z}}_{i}+s_{i}{\dot{s}}_{i}\\ & =z_{i}\left[-k_{q,i}z_{i}+M_{i}\left(s_{i}+{\tilde{\nu }}_{i}\right)\right]+s_{i}\left[-k_{\nu ,i}s_{i}-M_{i}z_{i}\right]\\ & =-k_{q,i}z_{i}^{2}-k_{\nu ,i}s_{i}^{2}+M_{i}z_{i}{\tilde{\nu }}_{i}. \end{array}$$

Since the tracking error evolves inside the funnel boundary, $M_{i}$ is bounded in the compact set, namely $0<M_{i}\leq {\bar{M}}_{i}$, where ${\bar{M}}_{i}$ is a positive constant. Using Young’s inequality, one has

$$M_{i}z_{i}{\tilde{\nu }}_{i}\leq \frac{{\bar{M}}_{i}}{2}z_{i}^{2}+\frac{{\bar{M}}_{i}}{2}{\tilde{\nu }}_{i}^{2}.$$

Therefore,

$${\dot{V}}_{i}\leq -\left(k_{q,i}-\frac{{\bar{M}}_{i}}{2}\right)z_{i}^{2}-k_{\nu ,i}s_{i}^{2}+\frac{{\bar{M}}_{i}}{2}{\tilde{\nu }}_{i}^{2}.$$

Let $\mu_{i}=\min\left\{k_{q,i}-{\bar{M}}_{i}/2,k_{\nu ,i}\right\}$. If $k_{q,i}>{\bar{M}}_{i}/2$ and $k_{\nu ,i}>0$, then $\mu_{i}>0$, and the following inequality holds:

(48) $${\dot{V}}_{i}\leq -2\mu_{i}V_{i}+\frac{{\bar{M}}_{i}}{2}{\tilde{\nu }}_{i}^{2}.$$

According to the ET-ESO estimation result, the velocity estimation error satisfies $\left|{\tilde{\nu }}_{i}\right|\leq \Theta_{i}h_{i}^{-2}$, where $\Theta_{i}$ is a positive constant. Substituting this bound into (48) yields

$${\dot{V}}_{i}\leq -2\mu_{i}V_{i}+\frac{{\bar{M}}_{i}}{2}\Theta_{i}^{2}h_{i}^{-4}.$$

Solving the above inequality gives

$$V_{i}\left(t\right)\leq V_{i}\left(0\right)e^{-2\mu_{i}t}+\frac{{\bar{M}}_{i}\Theta_{i}^{2}}{4\mu_{i}h_{i}^{4}}.$$

Thus, $z_{i}$ and $s_{i}$ are bounded. Since the transformed error $z_{i}$ is bounded, the original tracking error $e_{i}$ cannot reach the funnel boundary. Therefore, $|e_{i}\left(t\right)|<\rho_{i}\left(t\right)$ for all t≥0. This completes the proof. □

## 3. Simulation Results

To verify the effectiveness of the proposed event-triggered ESO-based prescribed-time funnel control method, numerical simulations are carried out in MATLAB/Simulink. The physical parameters of the micro quadrotor UAV are selected according to the Parrot Mambo mini-drone platform reported in [25]. This platform is a representative small-scale quadrotor and has been widely used for controller design, simulation validation, and real-time experimental studies. Therefore, it is suitable for evaluating the trajectory tracking and disturbance rejection performance of the proposed control strategy. The main physical parameters adopted in the simulations are summarized in Table 1. These parameters include the mass, arm length, gravitational acceleration, moments of inertia, thrust coefficient, and moment coefficient of the Parrot Mambo mini-drone, which are used to construct the six-degree-of-freedom micro quadrotor model in MATLAB/Simulink. The main design parameters of the proposed control algorithm are listed in Table 2. The PTFC parameters are used to determine the initial error boundary, steady-state accuracy, and prescribed convergence time. The ET-ESO parameters specify the observer bandwidths and event-triggered thresholds, while the controller gains are selected to regulate the convergence rates of the position, attitude, velocity, and angular velocity tracking errors.

To make the disturbance setting reproducible and to clarify the basis of the disturbance-suppression evaluation, the disturbance terms used in the simulations are explicitly specified. Since the proposed ESO treats external disturbances, unmodeled dynamics, damping uncertainties, and parameter perturbations as lumped disturbances, these effects are introduced into the translational and rotational acceleration channels as bounded time-varying lumped signals. The translational disturbance is written as

(49) $$\begin{array}{cc} d_{v}\left(t\right) & =d_{v,w}\left(t\right)+d_{v,a}\left(t\right)+d_{v,m}\left(t\right),\\ d_{v,w}\left(t\right) & =\left[\begin{array}{c} 0.35\sin(0.8t)+0.15\sin(2.1t)\\ 0.30\cos(0.6t)+0.12\sin(1.7t)\\ 0.25\sin(0.5t)+0.10\cos(1.5t) \end{array}\right],\\ d_{v,a}\left(t\right) & =\left[\begin{array}{c} -0.08V_{x}\left|V_{x}\right|\\ -0.08V_{y}\left|V_{y}\right|\\ -0.06V_{z}\left|V_{z}\right| \end{array}\right],\;d_{v,m}\left(t\right)=\left[\begin{array}{c} 0\\ 0\\ 0.05g\sin(0.4t) \end{array}\right]. \end{array}$$

Here, $d_{v,w}\left(t\right)$ represents the time-varying wind-induced acceleration, $d_{v,a}\left(t\right)$ represents the unmodeled aerodynamic drag-like effect, and $d_{v,m}\left(t\right)$ represents the equivalent acceleration uncertainty caused by mass perturbation. Similarly, the rotational disturbance is defined as

(50) $$\begin{array}{cc} d_{\omega }\left(t\right) & =d_{\omega ,w}\left(t\right)+d_{\omega ,a}\left(t\right)+d_{\omega ,J}\left(t\right),\\ d_{\omega ,w}\left(t\right) & =\left[\begin{array}{c} 0.08\sin(1.2t)\\ 0.07\cos(0.9t)\\ 0.05\sin(0.7t) \end{array}\right],\\ d_{\omega ,a}\left(t\right) & =\left[\begin{array}{c} -0.015\omega_{\phi }\left|\omega_{\phi }\right|\\ -0.015\omega_{\theta }\left|\omega_{\theta }\right|\\ -0.010\omega_{\psi }\left|\omega_{\psi }\right| \end{array}\right],\;d_{\omega ,J}\left(t\right)=\left[\begin{array}{c} 0.010\sin(0.5t)\\ 0.010\cos(0.5t)\\ 0.008\sin(0.3t) \end{array}\right]. \end{array}$$

$d_{\omega ,w}\left(t\right)$ denotes the wind-induced torque disturbance expressed in the angular-acceleration channel, $d_{\omega ,a}\left(t\right)$ represents the unmodeled aerodynamic damping effect, and $d_{\omega ,J}\left(t\right)$ denotes the equivalent uncertainty caused by inertia perturbation. All these disturbance components are bounded and smooth, which is consistent with Assumption 1. Therefore, the simulation evaluates disturbance estimation and compensation under explicitly defined time-varying disturbances rather than under an unspecified perturbation input.

**Figure 3:**
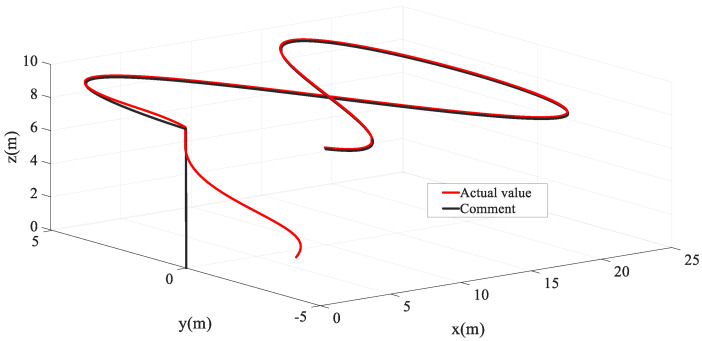
Figure-eight trajectory tracking performance.

**Figure 4:**
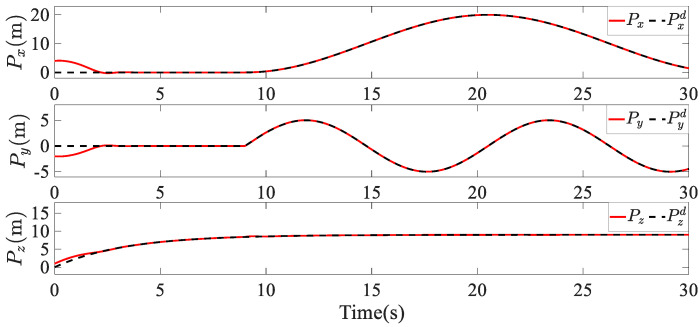
Single-axis position tracking responses.

**Figure 5:**
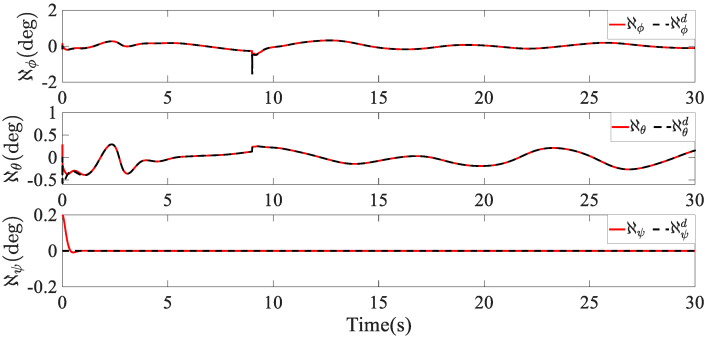
Roll, pitch, and yaw angle tracking responses.

**Figure 6:**
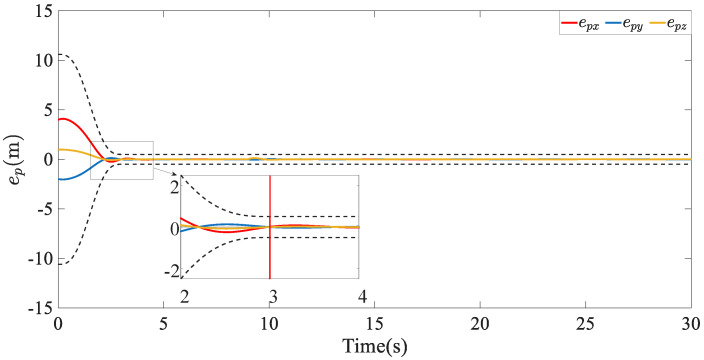
Position tracking errors under PTFC constraints.

**Figure 7:**
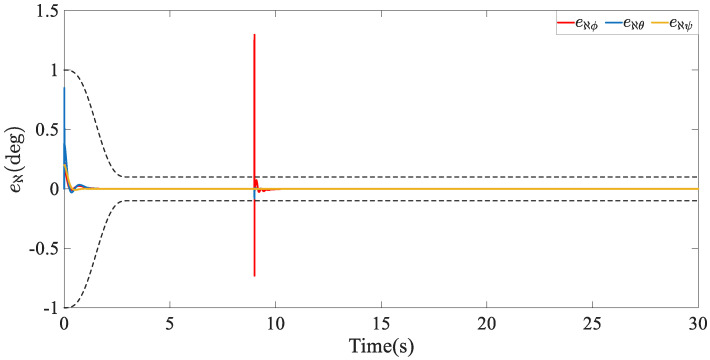
Attitude angle tracking errors under PTFC constraints.

**Figure 8:**
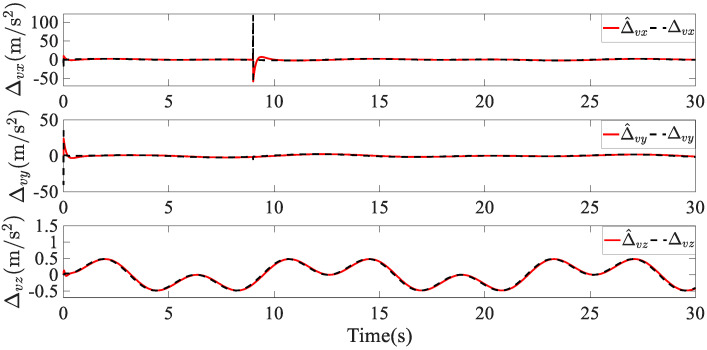
ET-ESO estimation of lumped disturbances in the translational subsystem.

**Figure 9:**
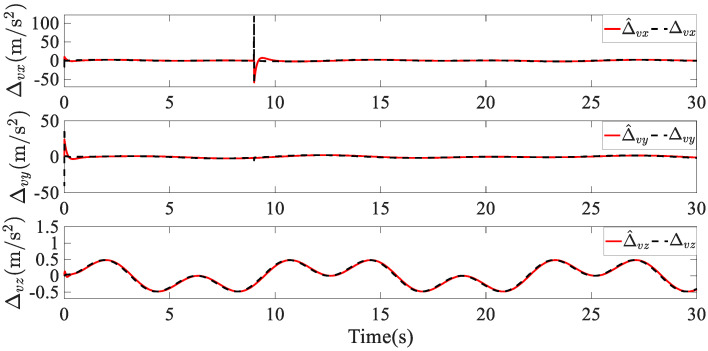
ET-ESO estimation of lumped disturbances in the rotational subsystem.

*Case 1: (Effectiveness certification of the presented controller):* As shown in Figure 3, Figure 4, Figure 5, Figure 6, Figure 7, Figure 8 and Figure 9, the trajectory tracking performance of the micro quadrotor UAV under the proposed control strategy is verified. Figure 3 presents the overall three-dimensional trajectory tracking result, from which it can be observed that the micro quadrotor UAV successfully follows the figure-eight reference trajectory. Figure 4 and Figure 5 show the single-axis position tracking responses and the attitude angle responses, respectively. The results indicate that, under the proposed control strategy, the UAV can smoothly track the reference signals within the prescribed time of 3 s while maintaining satisfactory dynamic response performance.

More specifically, Figure 3, Figure 4 and Figure 5 indicate that the proposed controller achieves coordinated position and attitude tracking for the micro quadrotor UAV. Since the translational motion of a quadrotor is indirectly generated through attitude adjustment and total thrust regulation, the smooth position responses imply that the virtual control law and the actual attitude control law work cooperatively. The bounded attitude responses also confirm that the rotational subsystem can provide sufficient attitude regulation for the translational tracking task.

Figure 6 and Figure 7 illustrate the convergence of the position tracking errors and attitude angle tracking errors under the prescribed-time funnel constraints, respectively. It can be observed that both the position errors and attitude errors converge within 3 s with small overshoots while remaining inside the predefined performance boundaries. This demonstrates that the proposed prescribed-time funnel control method can effectively constrain the transient response and improve the steady-state tracking accuracy. In addition, according to the designed flight mission, the UAV completes take-off and starts to perform the figure-eight trajectory tracking task at t=9 s. At this moment, relatively large transient attitude deviations occur. Nevertheless, under the regulation of the proposed controller, the attitude errors rapidly reconverge to the prescribed boundary region, indicating favorable dynamic recovery capability and robustness.

These results further verify the role of the prescribed-time funnel constraints. The tracking errors remain inside the predefined performance boundaries and converge to the desired accuracy region within the prescribed time. Therefore, the proposed PTFC not only improves steady-state accuracy, but also explicitly regulates transient performance, including convergence time and allowable overshoot. The relatively large attitude variation at the start of the figure-eight maneuver is mainly caused by the rapid change of the reference trajectory, while the fast return of the attitude errors to the prescribed boundary region demonstrates the transient recovery capability of the closed-loop system.

Figure 8 and Figure 9 present the estimation results of the ET-ESO for the lumped disturbances in the translational and rotational channels, respectively. The reference disturbance curves in these figures correspond to the explicitly defined disturbance inputs in Equations (49) and (50). The results show that, even with a reduced sampling and transmission frequency of state information, the proposed ET-ESO can still achieve satisfactory disturbance estimation performance. Therefore, the ET-ESO provides effective support for disturbance compensation in the proposed control framework.

In addition, Figure 8 and Figure 9 show that the ET-ESO can reconstruct the main trends of the translational and rotational lumped disturbances under event-triggered sampling. Although the observer uses event-triggered sampled signals instead of continuously updated measurements, the estimation errors remain bounded and do not destroy the prescribed-time tracking performance. The small residual estimation errors also explain the minor tracking deviations observed in the position and attitude responses. This observation is consistent with the theoretical analysis, where the ET-ESO estimation errors are proved to be practically bounded and are incorporated into the closed-loop stability proof.

**Figure 10:**
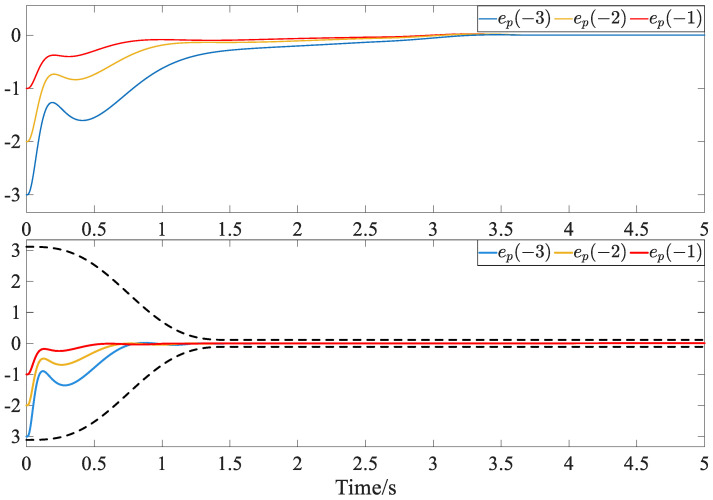
Comparison of PTFC and finite-time control strategy [26].

*Case 2: (Comparison with the finite-time control method in [26])*: This case is conducted to demonstrate the advantage of the proposed PTFC method over the finite-time control scheme reported in [26] in terms of prescribed-time stability. To ensure a fair comparison, the control parameters of all methods are carefully tuned to achieve their best possible performance. In this case, the prescribed convergence time is set as 1.5 s. As shown in the upper subplot of Figure 10, under the finite-time control method, the convergence behavior of the tracking error is significantly affected by the initial condition. The simulation results indicate that the settling time is strongly correlated with the magnitude of the initial tracking error. In other words, a larger initial deviation generally leads to a longer convergence time. Such initial-condition-dependent convergence may be insufficient for micro quadrotor UAV applications with strict time constraints, and additional parameter tuning or modification of the control law may be required to satisfy a given convergence-time requirement. In contrast, the lower subplot of Figure 10 shows that the proposed PTFC method guarantees that the tracking error converges to the desired region within the prescribed time of 1.5 s under different initial error conditions. Moreover, the smooth funnel error transformation adopted in this paper avoids the potential singularity problem associated with conventional error transformations, while maintaining satisfactory tracking accuracy. These results demonstrate that the proposed PTFC strategy provides an effective control framework that ensures explicit convergence-time guarantees, improved transient performance, and high-accuracy trajectory tracking under different initial conditions.

This comparison further shows the difference between finite-time convergence and prescribed-time performance. The finite-time control strategy can achieve convergence, but its settling behavior is closely related to the initial tracking error and controller gains. In contrast, the proposed PTFC imposes an explicit prescribed-time performance boundary, so the tracking error converges to the desired region within the predefined time under different initial conditions. This property is important for micro quadrotor UAVs performing time-critical missions, where the convergence time should be specified according to mission requirements rather than indirectly adjusted through gain tuning.

*Case 3: (Comparison between the proposed ET-ESO and conventional ESO [27])*: This case is conducted to evaluate the effectiveness of the proposed ET-ESO in reducing the sampling and information transmission burden. For comparison, the disturbance estimation performance of the proposed ET-ESO and the conventional continuously/periodically sampled ESO is investigated under the same disturbance conditions and simulation time. The simulation results show that the conventional ESO [27] requires 30,000 state sampling and updating operations during the whole simulation process, whereas the proposed ET-ESO only triggers 1084 sampling updates. This means that the number of updates required by the ET-ESO is only about 3.61% of that required by the conventional ESO, corresponding to a reduction of approximately 96.39% in sampling and information transmission. Therefore, the event-triggered mechanism can effectively avoid unnecessary continuous state acquisition and significantly reduce the sensor-to-controller communication burden. In terms of disturbance estimation accuracy, the estimation error variance of the conventional ESO is 5.3619, while that of the proposed ET-ESO is 6.1758. Although the estimation error variance of the ET-ESO is slightly larger than that of the conventional ESO, it still maintains comparable disturbance estimation accuracy. Overall, the proposed ET-ESO greatly reduces the sampling update frequency at the cost of only a slight degradation in estimation accuracy, indicating its potential applicability to resource-constrained micro quadrotor UAV platforms.

It should be noted that the resource-saving property of the proposed ET-ESO is evaluated at the algorithmic and simulation levels in this work. The ET-ESO only involves first-order observer state updates, threshold comparison, and basic algebraic operations, without neural network adaptation, fuzzy-rule updating, online optimization, or matrix inversion. Therefore, its computational complexity is relatively low and increases linearly with the number of controlled channels. For the six-degree-of-freedom micro quadrotor considered here, the observer only stores the estimated velocity/angular velocity states, the estimated lumped disturbances, and the latest event-triggered sampled signals for the translational and rotational subsystems. In the simulation comparison, the proposed ET-ESO reduces the number of sampling updates from 30,000 to 1084, corresponding to a reduction of 96.39% in sensor-to-controller information transmission. However, this result should be interpreted as a simulation-based update-frequency evaluation rather than a direct real-time benchmark on an embedded controller. Real-controller implementation, processor-in-the-loop verification, and flight experiments will be conducted in future work to further evaluate the real-time computational performance of the proposed method.

Table 3 further reveals the trade-off introduced by the event-triggered mechanism. Compared with the conventional ESO, the proposed ET-ESO reduces the number of sampling updates from 30,000 to 1084, corresponding to a 96.39% reduction in sensor-to-controller information transmission. The estimation error variance increases slightly from 5.3619 to 6.1758, which indicates that the communication reduction is achieved at the cost of only a minor loss of estimation accuracy. Therefore, the proposed method provides a practical balance among disturbance estimation accuracy, tracking performance, and update-frequency reduction.

## 4. Conclusions

In this paper, an ET-ESO-based PTFC method was proposed for robust trajectory tracking of micro quadrotor UAVs subject to lumped disturbances. The translational and rotational uncertainties, including wind disturbances, unmodeled aerodynamic effects, damping uncertainties, and parameter perturbations, were treated as lumped disturbances and estimated by two event-triggered ESOs. Compared with continuously sampled ESO schemes, the proposed event-triggered mechanism reduces unnecessary sensor-to-controller information transmission while maintaining disturbance estimation capability. Furthermore, a PTFC was developed to constrain the position and attitude tracking errors within predefined performance boundaries and ensure convergence to the desired accuracy region within a user-specified time. Lyapunov-based analysis proved the boundedness of all closed-loop signals and the validity of the prescribed funnel constraints. MATLAB/Simulink simulations based on the Parrot Mambo mini-drone parameters verified that the proposed method achieves effective disturbance compensation, improved transient tracking performance, and reduced control update frequency. The simulation results indicate that the proposed method can reduce the sampling update frequency while maintaining satisfactory disturbance estimation and tracking performance. Future work will include actuator saturation, motor dynamics, communication latency and delay compensation, processor-in-the-loop tests, embedded controller implementation, real-flight experiments, and possible integration with Tube-MPC-based constraint handling to further improve its applicability to practical networked micro quadrotor platforms.

## Figures and Tables

**Table 1:** Physical parameters of the Parrot Mambo mini-drone used in simulations.

Parameter	Symbol	Value	Unit
Mass	*m*	0.068	kg
Arm length	*l*	0.062	m
Gravitational acceleration	*g*	9.81	m/s^2^
Moment of inertia around *x*-axis	$I_{x}$	$6.86\times 10^{-5}$	kg·m^2^
Moment of inertia around *y*-axis	$I_{y}$	$9.20\times 10^{-5}$	kg·m^2^
Moment of inertia around *z*-axis	$I_{z}$	$1.366\times 10^{-4}$	kg·m^2^
Thrust coefficient	$k_{F}$	0.01	N/(rad^2^/s^2^)
Moment coefficient	$k_{M}$	$7.8263\times 10^{-4}$	N·m/(rad^2^/s^2^)

**Table 2:** Parameters of the proposed control algorithm.

Section	Parameters
PTFC	$\rho_{p,0}=10.6$, $\rho_{p,\infty }=0.5$, $T_{v}=T_{\omega }=3$ s, $\rho_{ℵ,0}=1$, $\rho_{ℵ,\infty }=0.1$.
ET-ESO	$\gamma_{\phi }=\gamma_{\theta }=\gamma_{\psi }=10$, $\varpi_{x}=\varpi_{y}=\varpi_{z}=20$, $\delta_{v}=\delta_{\omega }=0.001$.
Controller	$k_{px}=k_{py}=k_{pz}=3$, $k_{vx}=k_{vy}=k_{vz}=3$, $k_{ℵ\phi }=k_{ℵ\phi }=k_{ℵ\phi }=6$, $k_{\omega \phi }=k_{\omega \phi }=k_{\omega \phi }=12$.

**Table 3:** Comparison between the conventional ESO [27] and the proposed ET-ESO.

Method	Sampling Updates	Reduction Ratio	Error Variance
Conventional ESO [27]	30,000	–	5.3619
Proposed ET-ESO	1084	96.39%	6.1758

## Data Availability

The data presented in this study are available from the corresponding author upon reasonable request. The data have not been deposited in a public repository.
